# Development of Efficient Expression Systems for Bacteriolytic Proteases L1 and L5 of *Lysobacter capsici* XL1

**DOI:** 10.3390/ijms26136056

**Published:** 2025-06-24

**Authors:** Irina Kudryakova, Alexey Afoshin, Elena Leontyevskaya, Natalia Leontyevskaya

**Affiliations:** Laboratory of Microbial Cell Surface Biochemistry, G.K. Skryabin Institute of Biochemistry and Physiology of Microorganisms, FRC PSCBR, Russian Academy of Sciences, 5 Prosp. Nauki, 142290 Pushchino, Russia; kudryakovairina@yandex.ru (I.K.); alex080686@mail.ru (A.A.); ealeont@gmail.com (E.L.)

**Keywords:** efficient homologous expression system, bacteriolytic proteases, scaling up of the cultivation of expression strains, *Lysobacter capsici*

## Abstract

Secreted bacteriolytic proteases L1 and L5 of the Gram-negative bacterium *Lysobacter capsici* XL hydrolyze peptide bridges in bacterial peptidoglycans. Such specificity of action determines the prospects of these enzymes for medicine with the view of creating new antimicrobial drugs to combat antibiotic-resistant strains of pathogens. This research concerns the development of successful expression systems for producing active enzymes L1 and L5 in sufficient amounts for comprehensive studies. Based on *L. capsici* XL strains with deletions in the *alpA* (enzyme L1) and *alpB* (enzyme L5) genes and the constructed expression vectors pBBR1-MCS5 P_T5_–*alpA* and pBBR1-MCS5 P_T5_–*alpB*, we obtained expression strains *L. capsici* P_T5_–*alpA* and *L. capsici* P_T5_–*alpB*, respectively. The yields of enzymes L1 and L5 in the developed strains increased by 4 and 137 times, respectively, as compared to the wild-type strain. The cultivation of the expression strains was successfully scaled up under non-selective conditions in a 10-L bioreactor. After fermentation, the yields of enzymes L1 and L5 were 35.48 mg/L and 57.11 mg/L, respectively. The developed homologous expression systems of bacteriolytic proteases L1 and L5 have biotechnological value as compared to those obtained by us earlier based on heterologous expression systems, which have lower yields and labor-intensive purification schemes.

## 1. Introduction

The first bacteriolytic enzyme, lysozyme, was discovered by Alexander Fleming seven years before he discovered penicillin [1]. Lysozyme, as a less efficient agent, was temporarily forgotten for biomedicine until the problem of antibiotic resistance development in pathogenic microorganisms was understood. Today, this problem is of a global nature, and scientists continue a desperate search for antimicrobial agents. Bacteriolytic enzymes, as such agents, are again of interest for research as a basis for creating new antimicrobial drugs. However, lysozyme remains the only widely used bacteriolytic enzyme, although other such enzymes are known, including those that surpass it in antimicrobial activity. The use of other known bacteriolytic enzymes in practice is complicated by problems of their production on a biotechnologically significant scale.

Bacteriolytic enzymes are hydrolases that act on various bonds in the peptidoglycan of bacteria, including antibiotic-resistant strains of pathogens. Depending on the type of bond hydrolyzed in the peptidoglycan, one distinguishes between bacteriolytic proteases, amidases and glycosidases. For 60 years, our group has been studying extracellular bacteriolytic enzymes of the Gram-negative bacterium *Lysobacter capsici*. This bacterium is a unique producer of such enzymes, and to date, we have isolated and characterized to varying degrees 13 bacteriolytic enzymes. Moreover, thanks to transcriptomic and proteomic studies, a pool of genes of enzymes with putative bacteriolytic activity has been identified in this bacterium [2,3]. Based on the culture fluid of *L. capsici* XL1, a complex antimicrobial drug, lysoamidase, has been developed for the treatment of external infections. Today, we are developing antimicrobial drugs based on individual bacteriolytic enzymes, including homologous bacteriolytic proteases L1 (WND78730.1) and L5 (WND78729.1), which, according to the MEROPS database [4] belong to the family S1 (chymotrypsin family), subfamily S1D, subclan PA(S). In the cytosol of *L. capsici* cells, these enzymes are synthesized as preproproteins and are secreted outside the cell, passing through two membranes and transforming into mature enzymes. The identity of the primary sequences of the mature forms is 63%, and the overall structure is nearly identical (the root mean square deviation calculated at the superposition on the structures of these proteins by Cα atoms is 0.64 Å). The spatial structures of L1 and L5 have been resolved at 1.35 Å and 1.60 Å, respectively [5,6]. The optimal conditions for the manifestation of their bacteriolytic activities are slightly different (for L1 and L5, respectively): pH 7–11 and pH 7.5; 50 mM and 10 mM concentrations of buffer Tris-HCl; and optimum temperatures of 70 °C and 80 °C. Both enzymes hydrolyze bacterial cells and yeasts and also have proteolytic activities on casein and synthetic peptides [7,8]. These results indicate that enzymes L1 and L5 are promising for creating antimicrobial drugs with proteolytic and bacteriolytic action for the treatment of external infections.

As already noted, a sufficient amount of target protein is required to solve practical problems. It is the lack of successful expression systems that significantly complicates the study of bacteriolytic enzymes for biomedicine. Today, only a few expression systems are known for secreted bacteriolytic enzymes of Gram-negative bacteria: the α-lytic protease of *L. enzymogenes*, staphylolysin LasA of *Pseudomonas aeruginosa*, and pseudoalterin of *Pseudoalteromonas* sp. CF6-2 [9,10,11,12,13]. Earlier, we developed heterologous expression systems for enzymes L1 and L5, which enable the production of sufficient amounts of protein [14,15]. Most of the target enzymes, however, turned out to be inactive due to problems of refolding. It should be noted that the development of expression systems for secreted proteins of Gram-negative bacteria faces a number of difficulties. The fact is that such proteins become functionally active only after the entire path of topogenesis, from synthesis in the cytosol, secretion through the cytoplasmic membrane, the periplasmic stage (if any), and then secretion through the outer membrane. Each stage of topogenesis is necessary for correct maturation of protein. In some cases, protein helpers are required for terminal processing and conversion to a mature enzyme outside the cell. All these conditions cannot be created in heterologous expression systems. This was the way we came to the idea of expressing the genes of target enzymes in *Lysobacter*’s own cells. This is the first successful homologous expression system we have developed for the β-lytic protease of *L. capsici* [16]. The aim of this work was to develop homologous expression systems for bacteriolytic proteases L1 and L5, to scale up the process of cultivating the produced strains, and to assess the yield of target proteins.

## 2. Results

### 2.1. Production of a Strain with a Deletion of the alpA Gene

To express the genes of bacteriolytic proteases L1 and L5 in the native strain *L. capsici* XL1, it was necessary to construct expression plasmids and obtain the mutant strain *L. capsici* XL1Δ*alpA* with a deletion in the *alpA* gene. Mutant strain *L. capsici* XL1Δ*alpB* with a deletion in the *alpB* gene was also obtained by us earlier [17]. The use of mutant strains for expression of the *alpA* and *alpB* genes was necessary for an accurate assessment of the yields of target proteins.

**Figure 1:**
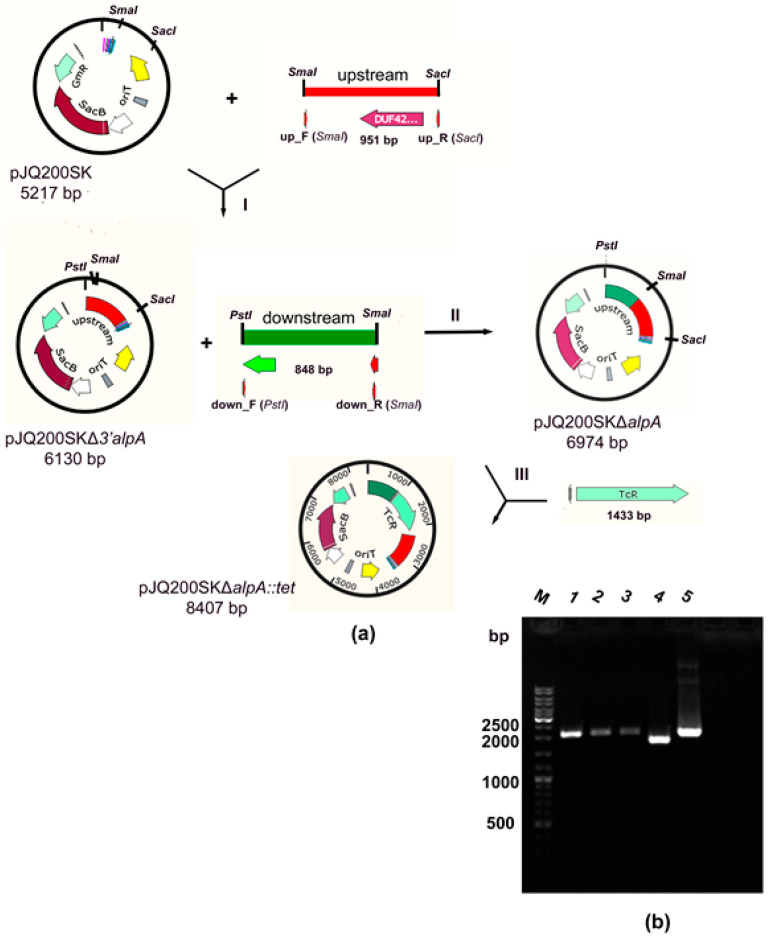
Production of mutant strain *L. capsici* XL1*ΔalpA*. (**a**) Scheme of constructing the vector pJQ200SK*ΔalpA::tet*. (**b**) Analysis of PCR products in 0.8% agarose gel: clones obtained by homologous recombination (1–3); DNA of *L. capsici* XL1 (4); plasmid pJQ200SK*ΔalpA::tet* (5).

A mutant strain of *L. capsici* XL1*ΔalpA* with a deletion in the *alpA* gene was obtained by homologous recombination. For this, the vector pJQ200SK*ΔalpA::tet* based on the suicide vector pJQ200SK was constructed (Figure 1a, Table 1 in Materials and Methods).

At the first stage, the plasmid pJQ200SKΔ3′*alpA* was produced by cloning in the suicide vector pJQ200SK a 951 bp fragment of DNA (of the 5′ end of the *alpA* gene and the adjacent upstream region), obtained as a result of amplification with *L. capsici* XL1 DNA and subsequently treated with restriction endonucleases *SmaI* and *SacI* (stage I, Figure 1a). At the second stage, the plasmid pJQ200SKΔ*alpA* was obtained by cloning an 848 bp DNA fragment (of the 3′ end of the *alpA* gene and the adjacent downstream region) amplified from *L. capsici* XL1 DNA and subsequently treated with restriction endonucleases *PstI* and *SmaI* (stage II, Figure 1a) into the pJQ200SKΔ3′*alpA* vector. At the last stage, the marked mutation of the tetracycline resistance (Tc^R^) gene cassette was introduced into the plasmid pJQ200SKΔ*alpA* treated with restriction endonuclease *SmaI* (stage III, Figure 1a). The Tc^R^ gene cassette was obtained by amplification from the plasmid pBR322. The oligonucleotide primers used in the work are given in Table 2 (Materials and Methods).

Thus, the plasmid pJQ200SK*ΔalpA::tet* was produced.

The plasmid pJQ200SK*ΔalpA::tet* was introduced into cells of *L. capsici* XL1 by electroporation. At the last stage of homologous recombination (Supplementary Materials, Figure S1), clones capable of growth on a nutrient medium with sucrose, resistant to tetracycline, and sensitive to gentamicin (Suc^R^Tc^R^Gm^S^ phenotype) were selected. This was indicative of successful recombination. Mutation was confirmed by PCR using selective primers (Table 2 of Materials and Methods). The size of the amplicons was found to be 2056 bp (Figure 1b), which corresponded to the replacement of the 1134 bp *alpA* gene region with the 1430 bp Tc^R^ gene cassette. The size of the obtained amplicons completely corresponded to that of the amplicon from the recombinant plasmid pJQ200SK*ΔalpA::tet.* The size of the amplicon from *L. capsici* XL1 DNA is 1756 bp, which corresponds to the full-length *alpA* gene with adjacent upstream and downstream fragments. The obtained results are indicative of a mutation in the *alpA* gene and, accordingly, of the successfully produced mutant strain *L. capsici* XL1*ΔalpA*.

### 2.2. Production of Expression Strains L. capsici P_T5_–alpB and L. capsici P_T5_–alpA

**Figure 2:**
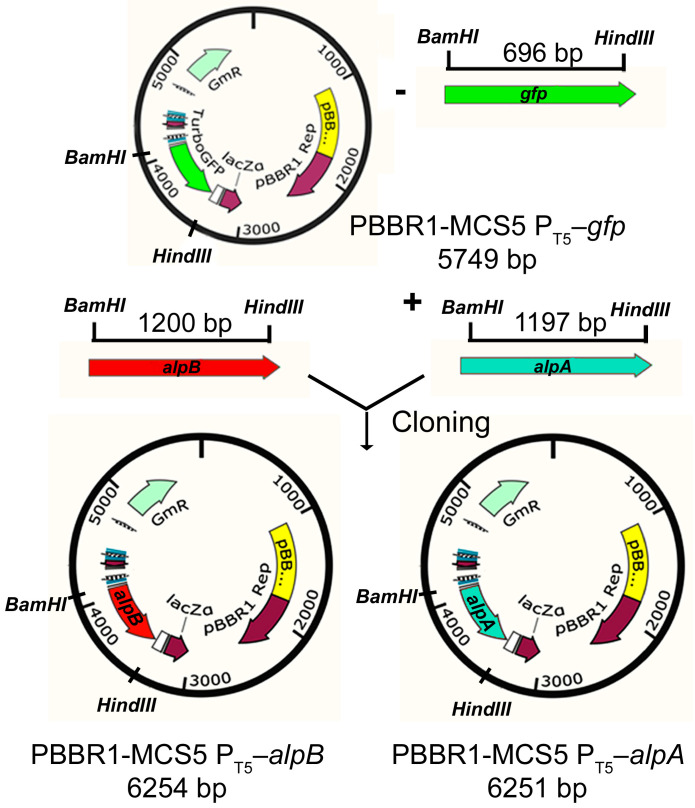
Scheme of constructing expression vectors pBBR1-MCS5 P_T5_–*alpB* and pBBR1-MCS5 P_T5_–*alpA* based on the plasmid pBBR1-MCS5 P_T5_–*gfp* with a Gm resistance marker.

Expression plasmids pBBR1-MCS5 P_T5_–*alpB* and pBBR1-MCS5 P_T5_–*alpA* were obtained by cloning the 1200 bp *alpB* (locus_tag = “RJ610_15605”) and 1197 bp *alpA* (locus_tag = “RJ610_15610”) genes, amplified with *L. capsici* XL1 DNA using selective primers (Table 2 of Materials and Methods) and subsequently treated with restriction endonucleases *BamHI* and *HindIII* (Figure 2) into the pBBR1-MCS5 P_T5_–*gfp* vector [16] that we had previously assembled.

As a result of electroporation of cells of *L. capsici* XL1*ΔalpB* and *L. capsici* XL1*ΔalpA* strains with plasmids pBBR1-MCS5 P_T5_–*alpB* and pBBR1-MCS5 P_T5_–*alpA*, respectively, we obtained expression strains *L. capsici* P_T5_–*alpB* and *L. capsici* P_T5_–*alpA*.

### 2.3. Expression and Purification of Bacteriolytic Proteases

**Figure 3:**
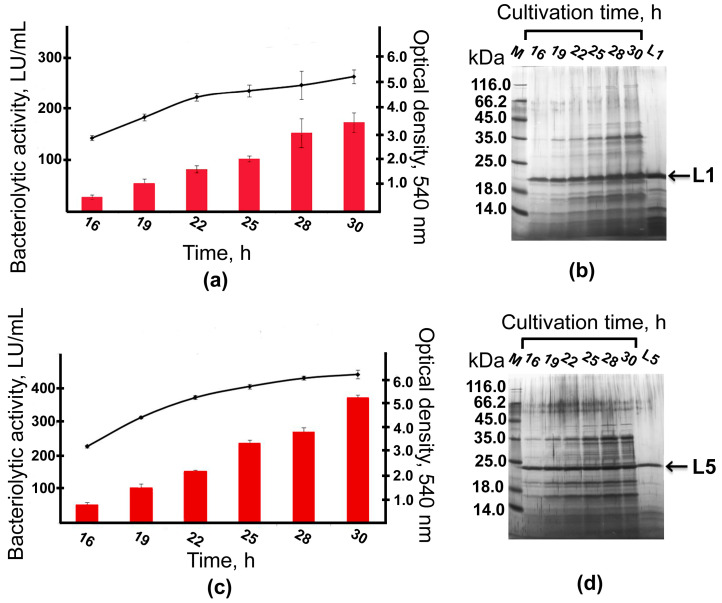
Dynamics of culture growth and bacteriolytic activity development of expression strains P_T5_–*alpA* (**a**) and P_T5_–*alpB* (**c**) during cultivation in flasks for 30 h. The results are presented as means ± SD. The values were obtained in two independent experiments each measured in triplicate. The bacteriolytic activity (LU/mL) was determined turbidimetrically using autoclaved cells of *Staphylococcus aureus* 209P. Electropherogram of proteins of the culture fluid of strains P_T5_–*alpA* (**b**) and P_T5_–*alpB* (**d**). M, a mix of protein standards. Markers of purified proteins L1 and L5 (0.5 µg and 0.7 µg, respectively).

Cells of strains P_T5_–*alpA* and P_T5_–*alpB* were cultivated on RM medium in flasks for 30 h. In the process of cultivation, the dynamics of culture growth and bacteriolytic activity development were studied (Figure 3).

Both strains were shown to reach the end of the exponential growth phase by 22 h of cultivation. No lysis was observed by 30 h of cultivation. The total bacteriolytic activity (total activity of bacteriolytic enzymes in the culture fluid) was observed already by 16 h of cultivation and continued to increase by 30 h to reach 171 LU/mL in the culture fluid of strain P_T5_–*alpA* (Figure 3a) and 371 LU/mL in the culture fluid of strain P_T5_–*alpB* (Figure 3c). Electrophoretic analysis showed that already by 16 h of cultivation, major protein bands observed in the culture fluid had mobility that coincided with the protein bands of the expressed bacteriolytic proteases L1 and L5 (Figure 3b,d).

**Figure 4:**
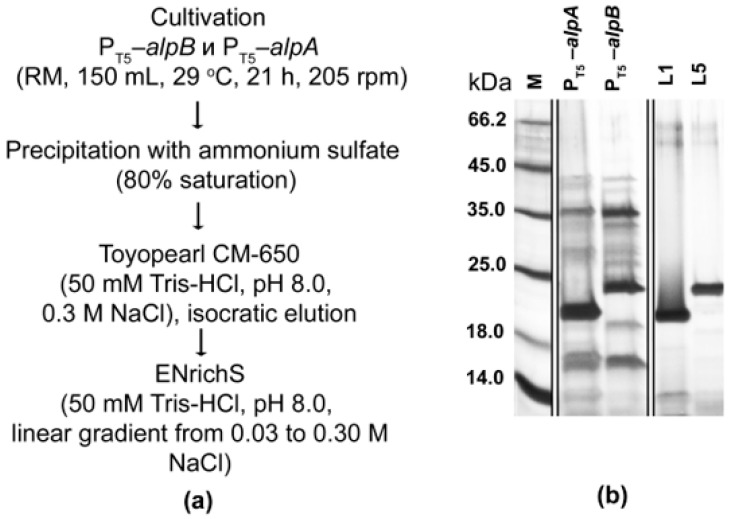
Purification of bacteriolytic proteases L1 and L5. (**a**) Purification scheme. (**b**) Electropherogram of culture fluid proteins (12 µL each) of expression strains (lanes 2 and 3). M, mix of protein standards. Proteins L1 and L5 (0.5 µg and 0.7 µg, respectively) after ENrichS (lanes 4 and 5). The original gel is presented in the Supplementary File Figure S2.

To retrieve bacteriolytic proteases L1 and L5 from the culture fluid of the expression strains, we developed a purification scheme that included protein precipitation with ammonium sulfate and two stages of cation exchange chromatography (Materials and Methods, Figure 4a).

Purification yielded preparations of bacteriolytic proteases L1 and L5 in an electrophoretically homogeneous state (Figure 4b) at concentrations of 0.195 ± 0.012 mg/mL and 0.371 ± 0.016 mg/mL, respectively. The yield was 18.98 ± 0.97 mg per liter of culture fluid for enzyme L1 and 27.44 ± 2.49 mg per liter for enzyme L5, which indicated the efficiency of the developed expression systems.

### 2.4. Maintenance of Recombinant Plasmids by Expression Strains P_T5_–alpA and P_T5_–alpB

**Figure 5:**
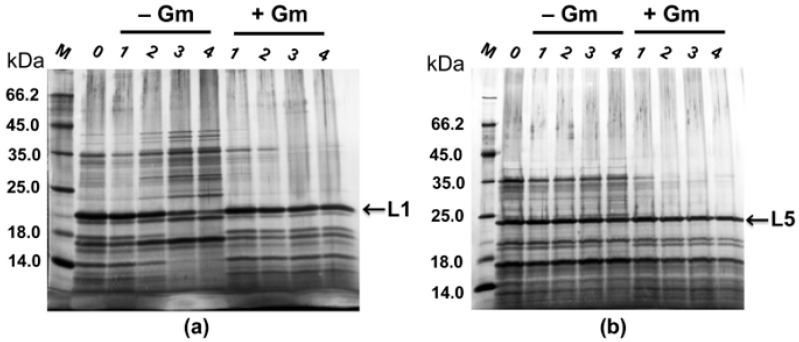
Electropherogram of culture fluid proteins during the cultivation of strains P_T5_–*alpA* (**a**) and P_T5_–*alpB* (**b**) under selective (+Gm) and non-selective conditions (−Gm). M, mix of protein standards. 0, culture fluid proteins after 21 h of cultivation (prior to the first passage); 1–4, passage number during cultivation, as carried out in two independent repeats.

Expression plasmids pBBR1-MCS5 P_T5_–*alpB* and pBBR1-MCS5 P_T5_–*alpA* (in accordance with Figure 2) carry the gene of Gm resistance. We investigated the ability of strains P_T5_–*alpB* and P_T5_–*alpA* to retain the expression plasmids when grown on RM medium under selective (in the presence of a selective factor, Gm) and non-selective (without Gm) conditions. The strains were cultivated for 21 h and then were reseeded into a new nutrient medium, continuing the cultivation under the same conditions. Four passages were made in this way. After 21 h, the culture fluid was analyzed electrophoretically (Figure 5).

As seen in Figure 5, both strains retain the expression plasmid over four passages, both under selective and non-selective conditions.

In the case of expression strain P_T5_–*alpA*, after the third passage under non-selective conditions (Figure 5a, passages 3 and 4), we observed a decrease in L1 production, which may indicate a partial elimination of the expression plasmid pBBR1-MCS5 P_T5_–*alpA*. However, only two passages without selective loading are needed to obtain the enzyme L1. Thus, cultivation of the expression strain under non-selective conditions will not affect the production process and final yields of the enzyme L1. These results indicate the possibility of producing bacteriolytic proteases L1 and L5 when cultivating expression strains on a medium without antibiotic, which is of great biotechnological significance.

### 2.5. Bioreactor Cultivation

**Figure 6:**
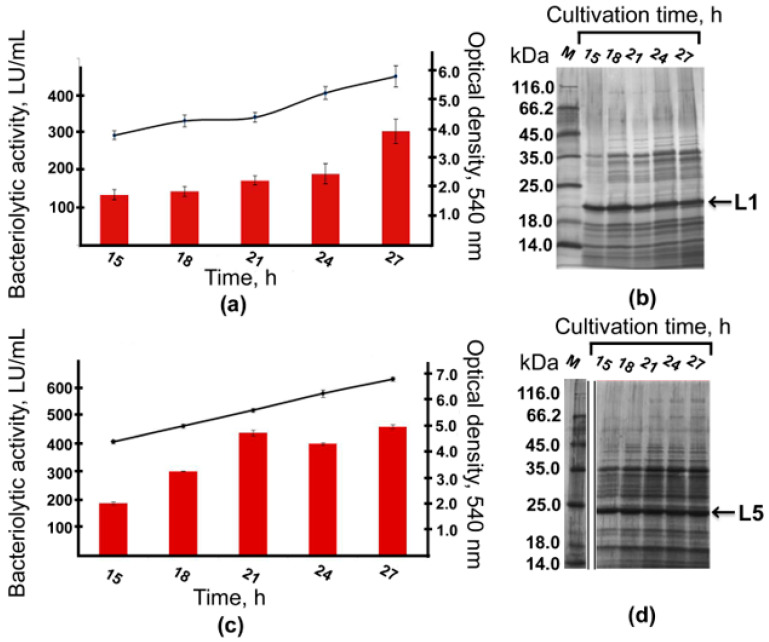
Cultivation of expression strains P_T5_–*alpA* and P_T5_–*alpB* in an ANKUM–2M bioreactor for 27 h. Dynamics of culture growth and bacteriolytic activity development of expression strains P_T5_–*alpA* (**a**) and P_T5_–*alpB* (**c**). The results are presented as means ± SD. The values were obtained in two independent experiments each measured in triplicate. The bacteriolytic activity (LU/mL) was determined turbidimetrically using autoclaved cells of *S. aureus* 209P. Electropherogram of proteins of the culture fluid of strains P_T5_–*alpA* (**b**) and P_T5_–*alpB* (**d**). M, mix of protein standards. Some 12 μL of the culture fluid preparation was added to the gel. The original gel is presented in Supplementary File Figure S3.

Cells of expression strains P_T5_–*alpA* and P_T5_–*alpB* were cultivated in an ANKUM–2M bioreactor with a working volume of 5 L on RM medium at 29 °C for 27 h without antibiotic Gm. During the fermentation process, the dynamics of growth and development of total bacteriolytic activity in the culture fluid were measured (Figure 6).

As seen in Figure 6, in both strains under fermentation conditions, the end of the exponential growth stage was observed by 24 h of cultivation. By the end of fermentation, the optical density of the cell culture of strain P_T5_–*alpA* was 5.9 o.u. (Figure 6a); that of strain P_T5_–*alpB* was 6.8 o.u. (Figure 6c), and no lysis was observed. The total bacteriolytic activity in the culture fluid of both strains was detected already by 15 h of cultivation. By the end of fermentation, the activity in the culture fluid of strain P_T5_–*alpA* was 305 LU/mL (Figure 6a); in the culture fluid of strain P_T5_–*alpB*, it was 457 LU/mL (Figure 6c). Electrophoretic analysis showed that by 15 h of cultivation, major protein bands observed in the culture fluid had already coincided (by their mobility) with the protein bands of the target enzymes L1 and L5 (Figure 6b,d).

The yield of enzymes was assessed after their purification from the culture fluid of the expression strains (27 h of cultivation) by the developed scheme (Figure 4a). As a result, the bacteriolytic proteases were obtained in an electrophoretically homogeneous state at a concentration of 0.247 ± 0.003 mg/mL and 0.420 ± 0.072 mg/mL, respectively. The specific bacteriolytic activity of enzyme L1 was 2785 ± 302 LU/mg; that of enzyme L5 was 1613 ± 302 LU/mg. The yield of enzyme L1 was 35.48 ± 0.31 mg per liter; that of enzyme L5 was 57.11 ± 1.28 mg per liter.

Thus, at this stage, the scaling up of the process of culturing expression strains showed the reproducibility of the results obtained under laboratory conditions in flasks.

## 3. Discussion

Bacteriolytic proteases L1 and L5 of *L. capsici* are promising agents for the creation of enzymatic antimicrobial drugs to treat external purulent infections. In this regard, the ability to produce these enzymes in quantities of biotechnological significance is an urgent task. The aim of this work was to develop efficient expression systems for enzymes L1 and L5. To obtain such systems, we used approaches we had previously developed for the expression of the β-lytic protease gene in *L. capsici*’s own cells [16]. As a result, we successfully developed expression systems for bacteriolytic proteases L1 and L5. The enzyme yields were 18.98 mg/L and 27.44 mg/L, respectively. These values are 4 and 137 times higher, respectively, than those of the wild-type strain (5.0 mg/L and 0.2 mg/L, respectively). The next important result was the successful scaling up of the expression strains’ cultivation process. As a result, the enzyme yields were 35.48 mg/L and 57.11 mg/L, respectively. The expression strains were also found to be stable when cultivated under non-selective conditions, which increases the biotechnological value of these systems. Now, we can produce target proteins for comprehensive studies, including the production of pilot batches of samples for preclinical and clinical trials.

Promising antimicrobial agents (enzymes, peptides, and antibiotics) sometimes do not enter practice due to a lack of biotechnologically significant expression systems or the impossibility of de novo synthesis; this is a serious problem. Our scientific interest is in the bacteriolytic enzymes of bacteria. At present, some expression systems for such enzymes are known (as detailed in Table 3).

**Table 3:** Expression systems for bacteriolytic enzymes.

Enzyme/Producing Strain	Expression Strain	Yield of Protein, mg/L	Refs
α-Lytic protease/ *Lysobacter enzymogenes*	*Escherichia coli* DG98	6.0	[9]
	*E. coli* TG1, JM109	77.0	[10]
	*Bacillus subtilis* DB104	14.0	[11]
Lysostaphin/ *Staphylococcus simulans* biovar *staphylolyticus*	*E. coli* TOP10	200.0	[18]
	*E. coli* BL21	55.0–70.0	[19]
	*Brevibacillus choshinensis*	90.0	[20]
	*B. subtilis* DSM402, *Lactobacillus casei* 102S	Not detected	[21]
	*Pichia pastoris* GS115	250.0	[22]
	*Lactococcus lactis* subsp. *cremoris* NZ3900	300.0	[23]
Staphylolysin/ *Pseudomonas aeruginosa*	*E. coli* JM109, *P. aeruginosa* FRD2128	Not detected	[12]
Enterolysin A/ *Enterococcus faecalis* II/1	*E. coli* SG13009	20.0	[24]
Zoocin A/ *Streptococcus equi* subspecies *zooepidemicus* 4881	*E. coli* M15	30.0	[25]
Pseudoalterin /*Pseudoalteromonas* sp. CF6-2	*Pseudoalteromonas* sp. SM20429	1.2	[13]

In the studies presented in Table 3, the researchers used different methods to estimate the enzyme yield, so it is difficult to make a fair comparison. One can note the high yields of lysostaphin from the Gram-positive bacteria *S. simulans* in heterologous expression systems. Based on the obtained yields, it could be concluded that these systems are successful. However, it should be noted that for bacteriolytic enzymes, the efficiency of the developed expression system is the amount of enzyme expressed in active units per unit of protein (LU/mg). As a result, the efficiency of the expression system for lysostaphin is difficult to assess, since LU/mg data in comparison with the native form of enzyme is not available. We assessed this problem when obtaining enzymes L1 and L5 from inclusion bodies [14,15]. The yields of enzymes L1 and L5 before renaturation were encouraging, at 64.5 mg/L and 65.5 mg/L, respectively. However, after renaturation, the yields were only 10% of the initial values. Comparison of the specific activities of the recombinant enzymes with the native ones showed that part of the recombinant protein was inactive. Similar results were obtained for recombinant enterolysin A: the specific activity was 8.5 times lower compared to native enterolysin A [24]. Earlier, we also developed expression strains for the enzymes L1 and L5 based on cells of *P. fluorescence* [26]. In this case, the yields of the enzymes were about 1 mg/L. Despite the fact that the Gram-negative bacterium *P. fluorescence* was used as the expression strain, the expression of the target proteins was not stable; culture lysis was observed. This once again confirms the importance of the natural topogenesis of secreted proteins of Gram-negative bacteria, which must be taken into account when developing expression systems for them. Table 3 also shows data for pseudoalterin from the Gram-negative bacterium *Pseudoalteromonas* sp. CF6-2. A homologous expression system has been developed for this enzyme to overcome the problems of autoprocessing in a foreign system. The protein yield was 1.2 mg/L [13]. However, those authors did not set the task of scaling up the expression strain cultivation process.

One of the main problems in the development of homologous expression systems is insufficient research into the genetics of bacteria-producing bacteriolytic enzymes. For example, the most investigated species in genetic terms among bacteria of the genus *Lysobacter* is *L. enzymogenes*. In this bacterium, the active promoters P_HSAF_ and P_GroEL_, including P_GroEL_ with a modification, have been studied [27,28]. Also, the CRISPR/dCas9-based transcription regulation system has been successfully used in *L. enzymogenes* cells [29]. These studies helped us begin the development of homologous expression systems for *L. capsici* enzymes. We are currently actively studying the genome of this bacterium in order to create highly efficient expression systems for its bacteriolytic enzymes.

Thus, our research suggests that, for extracellular bacteriolytic enzymes of Gram-negative bacteria, the use of homologous expression systems currently represents the most promising approach.

## 4. Materials and Methods

### 4.1. Strains, Plasmids, and Cultivation Conditions

The following strains and plasmids were used (Table 1).

**Table 1:** Strains and plasmids used.

Plasmids	Characteristics	Refs
**Plasmids**		
pJQ200SK	Suicide vector with the *sacB* gene, Gm^R^	[30]
pBR322	Origin of Tc^R^	[31]
pJQ200SKΔ3′*alpA*	pJQ200SK with the 5′ fragment of the *alpA* gene and the adjacent upstream region (951 bp)	This work
pJQ200SKΔ*alpA*	pJQ200SK with deletion in the *alpA* gene (1134 bp)	This work
pJQ200SK*ΔalpA::tet*	pJQ200SK with deletion in the *alpA* gene, marked by the Tc^R^ gene cassette	This work
pBBR1-MCS5 P_T5_–*gfp*	pBBR1-MCS5 with the *gfp* gene under control of bacteriophage T5 promoter	[16]
pBBR1-MCS5 P_T5_–*alpB*	pBBR1-MCS5 with the *alpB* gene under control of bacteriophage T5 promoter	This work
pBBR1-MCS5 P_T5_–*alpA*	pBBR1-MCS5 with the *alpA* gene under control of bacteriophage T5 promoter	This work
**Strains**		
*L. capsici* XL1	Wild-type	[32]
*L. capsici* XL1*ΔalpA*	Strain *L. capsici* XL1 with deletion in the *alpA* gene (1134 bp) and replacement of the corresponding segment by the Tc^R^ gene cassette	This work
*L. capsici* XL1*ΔalpB*	Strain *L. capsici* XL1 with deletion in the *alpB* gene (880 bp) and replacement of the corresponding segment by the Tc^R^ gene cassette	[17]
*L. capsici* P_T5_–*alpB*	Strain *L. capsici* XL1*ΔalpB* containing the plasmid pBBR1-MCS5 P_T5_–*alpB*	This work
*L. capsici* P_T5_–*alpA*	Strain *L. capsici* XL1*ΔalpA* containing the plasmid pBBR1-MCS5 P_T5_–*alpA*	This work
*Escherichia coli* XL1–Blue	*recA1 endA1 gyrA96 thi hsdR17 supE44* relA1 lac/[F’::Tn10 *proAB + lacI^q^ lacZDM15 traD36]*	[33]

Cells of *Lysobacter* strains were cultivated in 750 mL shake flasks containing 150 mL of LB-M nutrient medium (g/L): peptone, 5.0; yeast extract, 5.0; NaCl, 5.0, pH 7.5; or RM nutrient medium (g/L): glucose, 5.0; peptone, 2.0; yeast extract, 2.0; Na_2_HPO_4_ × 12H_2_O, 4.2; KH_2_PO_4_, 1.0; KCl, 0.6; MgSO_4_ × 7H_2_O, 5.0, pH 7.0 [32]. Cultivation was carried out on a Psu-20i orbital shaker (Biosan, Riga, Latvia) at 205 rpm and 29 °C. If necessary, antibiotic (Gm, 20 µg/mL) was added to the medium. Cells of *E. coli* XL1-Blue were grown on LB medium (g/L): tryptone, 10.0; yeast extract, 5.0; NaCl, 10.0, and pH 7.0 at 37 °C. Agarized nutrient media contained 1.5% agar.

### 4.2. Molecular–Genetic Manipulations

All molecular–genetic procedures were carried out in accordance with recommendations of reagent kits’ manufacturers and in correspondence with the manual of Sambrook and Russell [34]. Use was made of Q5 DNA polymerase (New England Biolabs, Ipswich, MA, USA), T4 DNA ligase, T4 polynucleotide kinase, and restriction endonucleases *BamHI*, *HindIII, PstI, SmaI, SacI* (Thermo Fisher Scientific, Waltham, MA, USA). To isolate DNA from agarose gel and bacterial cells, we used QIAquick gel extraction kit (Qiagen, Germantown, MD, USA) and diaGene kit (Diaem, Moscow, Russia), respectively. DNA was visualized at 302 nm using the GenoSens 2250 Touch gel documentation system (Clinx Science Instruments Co., Shanghai, China). The lengths of the separated DNA fragments were determined by SM0331 GeneRuler markers (Thermo Fisher Scientific, Waltham, MA, USA).

Highly competent cells of *E. coli* XL1-Blue were obtained by the RbCl method [35]. Transformation of expression plasmids into competent *Lysobacter* cells was carried out using the Lin method [36] with a modification. Approaches for introducing mutations into the genes of bacteriolytic enzymes by homologous recombination were developed by us earlier [17]. Briefly (Supplementary Materials, Figure S1), at the first stage, after the first crossing over, clones with the Suc^S^Tc^R^Gm^R^ phenotype were selected. Then, the selected clones were cultivated in LB-M medium at 29 °C to an optical density of 0.3 at 540 nm, followed by plating on LB-M medium containing 10% sucrose and 40 µg/mL Tc. As a result, clones with the Suc^R^Tc^R^Gm^S^ phenotype, which indicated the secondary crossing over, were selected.

The oligonucleotides used in the work are presented in Table 2. The oligonucleotides were designed using the SnapGene version 3.2.1 program and synthesized at the Evrogen facility (Moscow, Russia).

**Table 2:** Oligonucleotides used.

Oligonucleotides	Sequence	Goal
L5_*BamHI* (for)	GGATCCATGTCCGTATCGAAGTCGAATCTGC	To amplify the *alpB* gene (1200 bp) with DNA of *L. capsici* XL1
L5_*HindIII* (rev)	AAGCTTTCAACTCGTGACCAGGGCC	
L1_*BamHI* (for)	GGATCCATGTCCGTATCGAAGTCCAATGCG	To amplify the *alpA* gene (1197 bp) with DNA of *L. capsici* XL1
L1_*HindIII* (rev)	AAGCTTTCACGAGGTGACCAGGCTCAG	
T5_*KpnI* (for)	GGTACCGTGCCACCTGACGTCTAAG	To confirm the absence of mutations and the correct assembly of constructs
T5_XbaI (rev)	TCTAGACTGAAAATCTCGCCAAGCTAGC	
up_F (*SmaI*)	CCCGGGACTTCGATACTGACATGCG	To amplify the 951 bp fragment (of the 5′ end of the *alpA* gene and its upstream region) with DNA of *L. capsici* XL1
up_R (*SacI*)	GAGCTCGATTTCCGCCGCGATGG	
down_F (*PstI*)	CTGCAGCGCGGCTTCCTGG	To amplify the 848 bp fragment (of the 3′ end of the *alpA* gene and its downstream region) with DNA of *L. capsici* XL1
down_R (*SmaI*)	CCCGGGCCGATCCTGAGCC	
Tc (for)	GAATTCTCATGTTTGACAGCTTATCATCGA	To amplify the 1433 bp fragment of the Tc cassette from the plasmid pBR322
Tc (rev)	CCCGAGATGCGCCG	
check_F	CTCGATAAAGGCCACATC	To amplify the 2056 bp fragment from the plasmid pJQ200SK*ΔalpA::tet* and clones with deletion in the *alpA* geneAmplification of a 1756 bp fragment with DNA of *L. capsici* XL1
check_R	ACGGTTCATGTCCTTATG	

### 4.3. Purification of Bacteriolytic Proteases L1 and L5

Cells of expression strains P_T5_–*alpA* and P_T5_–*alpB* were cultivated on RM medium with Gm for 21 h (in flasks) and without Gm for 27 h (in bioreactor). Then, 300 mL of the culture was centrifuged at 7000× *g* for 20 min to separate cells. Proteins were precipitated from the resulting culture fluid with (NH_4_)_2_SO_4_ to 80% saturation at 4 °C and centrifuged at 22,470× *g* for 1 h. The protein pellet was suspended in 50 mM Tris-HCl, pH 8.0, and dialyzed against 100 V of the same buffer. Then, a purification scheme was developed. At the first stage, use was made of cation exchange chromatography on a Toyopearl CM-650 column (Merck, Darmstadt, Germany) equilibrated with 50 mM Tris-HCl, pH 8.0. Isocratic elution of proteins was performed with 50 mM Tris-HCl buffer, pH 8.0, containing 0.3 M NaCl. At the second stage, the protein preparation preliminarily dialyzed against 50 mM Tris-HCl, pH 8.0 was applied to an ENrichS column (Bio-Rad, Hercules, CA, USA) equilibrated with the same buffer and connected to the NGC chromatographic system (Bio-Rad, Hercules, CA, USA). Proteins were eluted with a linear gradient of NaCl from 0.03 to 0.30 M. Fractions with bacteriolytic activity and containing electrophoretically homogeneous bacteriolytic proteases L1 and L5 were pooled and stored at minus 20 °C.

### 4.4. Measurement of Bacteriolytic Activity by Turbidimetric Method

Autoclaved lyophilized cells of *S. aureus* 209P were used as a substrate to determine the bacteriolytic activity. The reaction mixture contained 975 µL of cell suspension with OD_540_ = 0.5 in 10 mM Tris-HCl, pH 8.0 and 25 µL of enzyme preparation (culture fluid of the expression strains or solutions of enzymes L1 and L5). The mixture was incubated at 37 °C for 5 min. The reaction was stopped by placing the tubes in an ice bath. The drop in the optical density of the suspension was recorded at 540 nm using a DU 730 spectrophotometer (Beckman, Brea, CA, USA). The bacteriolytic activity (LU/mL) was determined using the following formula:

[0.5 (OD_540_ of the control sample suspension) − OD5_40_ of the test sample suspension] × 1000 × L (total reaction volume) × dilution/[min (time of reaction) × L (volume of sample) × 0.01 (correction coefficient for the OD reduction per min)].

For homogeneous bacteriolytic proteases L1 and L5, the specific activity, LU/mg, was also calculated.

Bacteriolytic activity measurements were conducted in two independent experiments, each time in triplicate.

### 4.5. Sodium Dodecyl Sulfate–Polyacrylamide Gel Electrophoresis

Electrophoresis of proteins was carried out in 12.5% polyacrylamide gel in the presence of sodium dodecyl sulfate using the Laemmli method [37]. Culture fluid preparations (12 μL each) of the expression strains and purified bacteriolytic proteases L1 and L5 (0.5 μg and 0.7 μg, respectively), preheated in sample buffer at 99 °C for 10 min, were added to the gel. SM0431 GeneRulers (Thermo Scientific, Waltham, MA, USA) were used as molecular weight markers. Electrophoresis in a stacking gel was carried out at 90 V and in a separating gel at 180 V. Protein bands in the gel were revealed by staining with imidazole and ZnCl_2_ solutions [38].

### 4.6. Protein Concentration Assay

The concentration of bacteriolytic proteases L1 and L5 was measured by the Bradford method [39] using the Coomassie reagent (Thermo Fisher Scientific, Waltham, MA, USA). The reaction was carried out according to the manufacturers’ protocols. The protein concentration was determined by a calibration curve plotted for an aqueous solution of BSA (Sigma, Ronkonkoma, NY, USA) within the range of 1–25 µg/mL.

### 4.7. Fermentation

An ANKUM-2M bioreactor (Special Design Bureau for Biological Instrumentation, USSR Academy of Sciences, Pushchino) with a total volume of 10 L and a filling factor of 0.5 was used to cultivate the expression strains. The inoculum was grown on RM medium with Gm for 20 h. Fermentation of the cells of strains P_T5_–*alpA* and P_T5_–*alpB* was carried out on RM medium for 27 h at 29 °C, with a stirrer speed of 600 rpm and a dissolved oxygen concentration of 30%. The cultivation was carried out in two independent experiments.

## Data Availability

The original contributions presented in this study are included in the article/Supplementary Materials. Further inquiries can be directed to the corresponding author.

## Supplementary Materials

The following supporting information can be downloaded at: https://www.mdpi.com/article/10.3390/ijms26136056/s1.
