# Supplementary material for: Development of Efficient Expression Systems for Bacteriolytic Proteases L1 and L5 of Lysobacter capsici XL1
Source: Int J Mol Sci. 2025 Jun 24;26(13):6056. doi: 10.3390/ijms26136056 (PMC12250508; doi:10.3390/ijms26136056)
Supplement: Supplementary file 1 [file ijms-26-06056-s001.zip › ijms-3689400-supplementary.pdf]

# Development of Efficient Expression Systems for Bacteriolytic Proteases L1 and L5 of *Lysobacter capsici* XL1

**Irina Kudryakova, Alexey Afoshin, Elena Leontyevskaya and Natalia Leontyevskaya ***

Laboratory of Microbial Cell Surface Biochemistry, G.K. Skryabin Institute of Biochemistry and Physiology of Microorganisms, FRC PSCBR, Russian Academy of Sciences, 5 Prosp. Nauki, 142290 Pushchino, Russia; kudryakovairina@yandex.ru (I.K.); alex080686@mail.ru (A.A.); ealeont@gmail.com (E.L.)

* Correspondence: vasilyevanv@rambler.ru

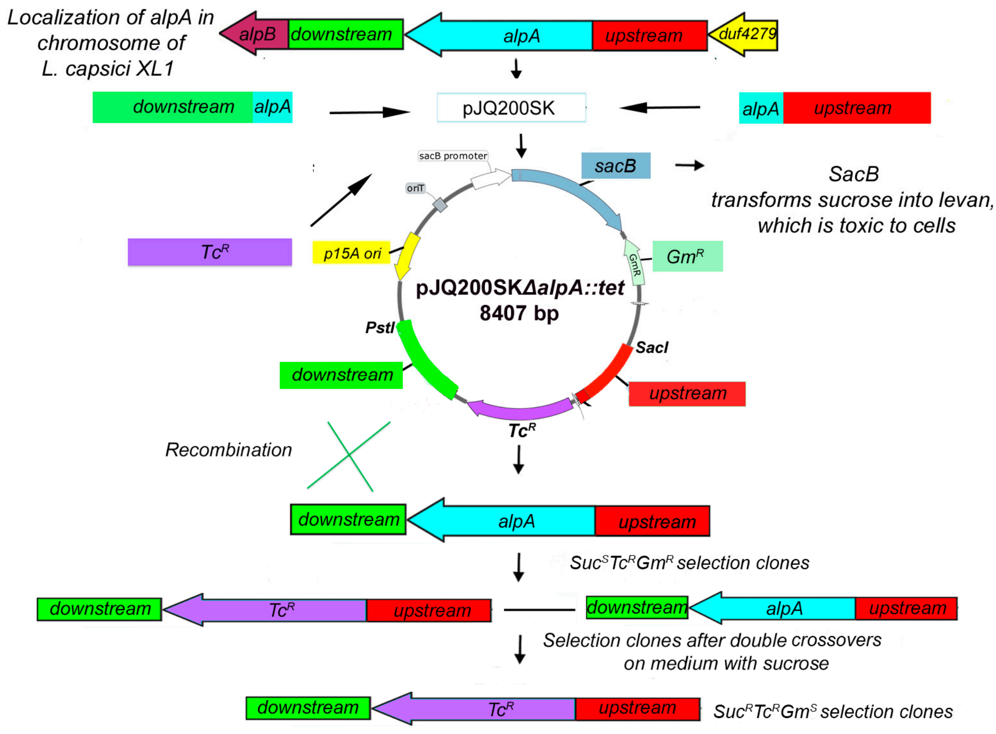


**Supplementary file Figure S1.** Introduction of a deletion into the *alpA* gene of *L. capsici* XL1 by homologous recombination.

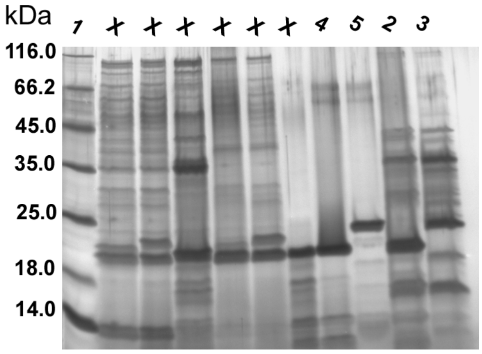


**Supplementary file Figure S2.** Original gel images for Figure 4b: lane 1 corresponds to M of Figure 4b; lanes 2 and 3, to samples of culture fluid of strains $P_{T5}$–*alpA* and $P_{T5}$–*alpB,* respectively, of Figure 4b; lanes 4 and 5, to samples of purified bacteriolytic proteases L1 and L5, respectively, of Figure 4b.

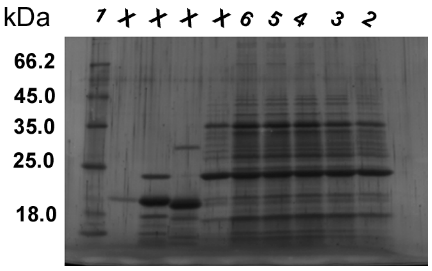


**Supplementary file Figure S3.** Original gel images for Figure 6d: lane 1 corresponds to M of Figure 6d; lanes 2–6, to samples of culture fluid of strain $P_{T5}$–*alpB* of Figure 6d.
